# Psychology of Wolf
*Opera J. C. Wolfii. Breslau.


**Published:** 1857-10-01

**Authors:** 


### Art. VIII.—PSYCHOLOGY OF WOLF.*

BY PROFESSOR HOPPUS, LL.D.

In the career of German psychical speculation, a conspicuous part was borne by Johann Christian von Wolf. He is mainly characterized as the author of a philosophy founded on that of Leibnitz, but modified, systematized, and exhibited, according to his own forms and modes of thinking, being expanded into elementary treatises, and reduced from the scattered and desultory materials which abound in the multifarious productions of Leibnitz, into a digested arrangement. Hence it has been usual on the Continent, since the time of Wolf, to designate the mass of these speculations as the Leibnitz-Wolfian philosophy.

Like not a few others of the great metaphysicians of the ideal school, from Descartes downwards, Wolf was a man of encyclopædic learning in his day. He was born at Breslau, in 1679, and early devoted himself to the eager pursuit of knowledge in the Magdalen Gymnasium of his native city. He here acquired no small reputation for his skill in the wrangling disputations which were the fashion of the age, and he is said to have met with few who could compete with him in these exercises. He had scarcely passed his youth, when having heard of the effects which the writings of Descartes were producing, in opposition to the old scholasticism still in vogue, Wolf ardently applied himself to the Cartesian philosophy; and he appears, at this early age, to have conceived the idea of doing for practical, the same service which Descartes had aimed to render to theoretical philosophy. This task he hoped to achieve by the application of a stricter

* Opera J. C. Wolfii. Breslau.

mathematical method than had hitherto been attempted. With this view, he entered on the study of the exact sciences.

In furtherance of his design, he repaired to Jena, where mathematics engrossed his attention, in the hope of preparing himself for securing a solid basis for metaphysics in the science of number and quantity. He afterwards passed some years at Leipzig ; and he here maintained a thesis on the mathematical method, which was published in 1781, under the title *Philosophia Practica, mathematico modo conscripta.* At Leipzig he studied under Tschirnhausen, delivered lectures, and published his treatises *De Rotis Dentatis*, and *De Algorithmo Infinitesimali Differentiali.* It appears to have been his early intention to take holy orders : but this project was overruled by his ardent desire for the pursuit of intellectual and scientific truth ; and perhaps in some measure from the encouragement he received not only from Tschirnhausen, but also from Leibnitz himself, to whom he had been introduced by Munken. Perhaps Leibnitz saw that Wolf was eminently adapted, by his methodical and rigorously logical cast of mind, to complete what Descartes and himself had done so much towards achieving—the final overthrow of the empire of Aristotle in Germany.

In 1711 our philosopher became professor of mathematics at Halle, having previously been invited to Giessen. At Halle he wrote his treatise *De Methodo Mathematicâ,* and his work entitled *Elementa Matheseos Universæ.* This voluminous publication, which in the later Genevan edition extended to five volumes quarto, comprises, along with other subjects, " A Description of the Mathematical Method ; Arithmetic ; Geometry ; Plane and Spherical Trigonometry ; Mechanics and Statics ; Hydrostatics ; Optics ; Perspective ; Astronomy ; Geography ; Chronology ; Dialling ; Pyrotechny ; Architecture ;" and an " Account of the Principal Writings of Mathematicians." In 1710 he was elected a Fellow of the Royal Society of London. In 1728 he published his " Tables of Sines, Tangents, and Logarithms." The " Acta Eruditorum" bear many testimonies to his industry and learning, in the papers which he contributed relating to the mathematical and physical sciences.

But Wolf's inclination led him to devote his energies almost entirely to the study of metaphysical and moral philosophy ; and in order to gain the public ear for these subjects, he did not hesitate to depart from the immemorial custom in Germany of publishing learned works in Latin. He made the German language the vehicle of his philosophy. This circumstance alone could not fail to give him a great advantage over Leibnitz and all who had preceded him in the career of speculation in that country ; no doubt it contributed very greatly to the popularity of his more strictly philosophical writings. His works in Latin and German are from sixty to seventy in number. Between the years 1712 and 1723, he published his *Vernünftige Gedanken von den Kräften des menschlichen Verstandes—Metaphysik oder Vernünftige Gedanken von Gott, der Welt, und der Seele des Menschen, auch allen Dingen überhaupt** — *Anmerkungen dazu—Versuche zur Erkentniss der Natur und Kunst—Vernünftige Gedanken von den Wirkungen der Natur—Von den Absichten der Natürlichen Dinge—Von des Menschen Thun und Lassen—Politik, oder vernünftige Gedanken von dem gesellschaftlichen Leben der Menschen—Nachricht von seinen eigenen Schriften in Deutscher Sprache in verschiedenen Theilen der Weltweisheit—Gesammelte Kleine philosophische Schriften.* Wolf also published at a later period a "Mathematical Dictionary," in German. The detached pieces in this language under the title of *Gedanken,* which treat of the powers of the human mind, the Deity, the universe, the operations of nature, the search after happiness, the constitution of society, and other subjects, present their respective doctrines in a simpler and more concise form than is adopted in the great work in Latin, which embraces the same topics in an extensive and far too diffuse course of philosophy. These smaller pieces which preceded are still highly useful to all who read German, and are calculated to give a very adequate knowledge of the author's doctrines; indeed it was these pieces in the vernacular tongue that spread the fame of Wolf over Germany.

Wolf was now regarded as Leibnitz's most illustrious disciple, and he received while at Halle invitations to the chairs of philosophy, at Wittenberg, Leipzig, and St. Petersburg. However, he remained at Halle till a violent hostility towards him arose among the theological professors of that university. Wolf himself was, at the time, Dean of the Faculty of Theology, and a jealousy appears to have arisen from the circumstance of the Dean appointing one of his own pupils to be his assistant, in preference to another student whom he thought incompetent, but who, unfortunately, happened to be the son of one of the other professors. Thus an occasion arose which proved of serious consequence to our hitherto prosperous philosopher. It is certain that Wolf was a man of pure morals, amiable temper, and orthodox Christianity. In conformity with his system of proving everything, he applied the Cartesian method strictly to religion, and he endeavoured to establish its usually admitted doctrines by a series of syllogistic demonstrations. He maintained that the truths of religion ought to be believed, because they could

* If ever there was a man who tried to grasp the *omne scibile,* it was surely Wolf!

be put to the test of the syllogism. His opponents held that they ought to be believed because they were received from Scripture by the almost universal consent of the Church. Wolf's intention was to do service to Christianity by reducing it to logic; the divines of that day were not prepared for so bold an innovation, and they charged him with heresy, and inferred everything which is always supposed to be at once capable of being inferred from such a charge. His philosophy was opposed to religion and morals—he substituted the agency of mechanical causes for the empire of Providence—he introduced fatalism into the events of the moral world—his views regarding "pre-established harmony" left no room for freedom in God or man. That these allegations were not just was no matter: they alarmed heads that did not want their brains puzzled with metaphysics; and which, perhaps, could not always, if they would, very well distinguish between a mere metaphysical question and the article of a creed.

To make matters worse, it happened that Wolf, in lecturing to his class,* had pronounced an eulogium on the moral precepts of Confucius, which had lately become known in the West by means of the researches of the Jesuit missionaries. This unlucky circumstance greatly inflamed the controversy. To approve of the ethical doctrines of a pagan philosopher was pronounced, in that bigoted age, unworthy of a Christian divine, and even heretical, by the dominant party at Halle. They were known in Germany by the name of "Pietists," and with all credit for sincerity, their views of religion, of man and his moral nature, were, in some respects, founded on narrow and mistaken conceptions and interpretations of Holy Scripture. Hermann Franke, founder of the Orphan School, one of the best and most celebrated of the Pietists, held at this time a theological chair in the University: crowds of students from various parts of Germany had flocked to hear him; but it was in vain that he opposed the new philosophy from his chair; he now found himself comparatively deserted for the more attractive lectures of Wolf. The Pietists first accused him before the Academical Senate, and afterwards complained of him to the King of Prussia, Frederic William I. They proceeded to the length of charging him with atheism. They denied the possibility of his demonstrations, though some of their party inconsistently adopted a line of argument very similar to that of Wolf himself.† It was even alleged by some of his opponents

* This Lecture was published, entitled *Oratio de Sinarum Philosophiâ.* Halle, 1726.

† Vid. Lange's *Causa Dei et Religionis Naturalis adversus Atheismum et Pseudo-philosophiam.* Halle: 1723.—Also Ribov's *Beweis dass die geoffenbarte Religion nicht könne aus der Vernunft erwiesen werden.* Göttingen: 1740.

that his doctrines were dangerous to the army, and tended to excuse desertion! This was enough: Wolf had orders to quit the Prussian dominions in two days, on pain of the severest penalties of the law; though he informed the minister at Berlin that he had intended to publish his lecture at Rome, with the consent of the Inquisition—so little did he apprehend persecution for it in philosophical and enlightened Germany! But such was the feeling among the Pietists, that no sooner had Wolf quitted Halle, than the excellent Franke threw himself on his knees in the church, and gave thanks to God for the deliverance, almost as though Wolf had been the animal which his name imports.

Driven thus summarily, and without a fair hearing, from Halle, in 1723, Wolf took up his abode in Hesse Cassell, where he was well received by the Landgrave, who appointed him Professor of Philosophy and Mathematics at Marburg, and conferred on him the title of Aulic Councillor. Wolf continued here about eighteen years, and from 1728 to 1740, he published his metaphysical works in the large Latin edition. This immense course of philosophical disquisition comprehends *Philosophia rationalis, sive Logica methodo scientifica pertractata—Psychologia empirica—Philosophia prima sive Ontologia—Cosmologia generalis—Psychologia rationalis—Theologia naturalis—Philosophia practica universalis—Philosophia moralis sive Ethica—Jus Naturæ.* He also wrote *Specimen Physicæ ad Theologiam naturalem applicatæ.* His Latin course of philosophy was published in no less than twenty-four quarto volumes. The *Jus Naturæ,* alone, in the Frankfort and Leipzig edition of 1732, is in eight quartos. His *Jus Gentium* was not published till 1752.

Wolf did not fail, in his new chair at Marburg, where he was beyond the reach of his opponents, to vindicate himself with a vehemence proportioned to their attacks. The dispute, indeed, had extended itself far and wide over Germany, and Wolfian fought with anti-Wolfian incessant metaphysical battles, in which neither party gained the victory, though both claimed it. Among his own followers the controversy was identified with the independence of philosophy. At length the opinion gained ground that Wolf had been unjustly and harshly dealt with; and that neither religion nor good government would be promoted by the attempt to suppress freedom of inquiry—an attempt which is always sure, sooner or later, to be succeeded by a re-action. Frederic appointed new commissioners to examine and report on the writings of the banished professor. They declared that Wolf's philosophy contained nothing opposed to the interests of the State, nothing dangerous to morals and religion, nothing contrary to the orthodox Lutheran doctrine. Wolf was repeatedly invited to return to Halle; this, however, he refused to do so long as Frederic William reigned. In 1740, Frederic the Great ascended the throne, and the next year Wolf accepted the invitation. Among other honours domestic and foreign which now fell to his lot, the king made him a privy-councillor, vice-chancellor and afterwards chancellor of the university; and the Elector of Bavaria conferred on him the dignity of a Baron of the Empire. Wolf died in 1754, of gout in the stomach, in the seventy-sixth year of his age, having borne his sufferings with Christian piety and fortitude.

From Descartes Wolf imbibed that independence of thought, and attachment to the mathematical method which characterized the school of that eminent philosopher. From Tschirnhausen he obtained the idea of the necessity of aiming at precision of language, logical exactness in definition, and the harmonious combination of the *à priori* method with the results of experience. But it was to Leibnitz that Wolf directed his principal attention. He thought the time was come for attempting a national philosophy to which Leibnitz might furnish the clue, more especially as Germany was now no longer contented with having a foreign literature, and was advancing towards one that should be indigenous to its own soil. Wolf found the thinking world grown weary of the scholastics. Aristotle, whose excellences and errors were equally confounded with the dogmas of the schoolmen, had already shared largely in their fate. Platonism was less known than Aristotelianism, and it was moreover always wanting in a didactic method. Thomasius was not elevated enough for the taste of the learned public. Descartes had not so applied his principles as to promise durable results. Leibnitz, indeed, the cynosure and prodigy of his age and nation, had laid foundations which the best minds of the day were disposed to regard as solid; but on these foundations he had only built limited and detached erections. Wolf had an ambition to finish what Leibnitz had begun, with such alterations both of plan and execution as he might himself deem expedient; and, whatever may have been his success or failure, he went to work like a true German, and fully sustained the character of a most indefatigable student, and a writer of vast industry and enormous labour. He appears to have had several points of character in which he much resembled the head of his school. He was candid, simple, and disinterested. When the King of Sweden asked him what he could do to serve him, he merely replied that he "wanted nothing." Amidst vicissitudes of fortune, and bigoted and unjust persecutions for his opinions, he sustained equality and serenity of mind. Like his great master in philosophy, he was not free from vanity; but he was ingenuous and urbane, and was generous even towards his enemies. The love of intellectual truth was a passion with him. He was more attached to method and system than Leibnitz, at least to its forms and signs. In originality, however, he was far inferior to his master, Leibnitz was the father of German philosophy: Wolf was the reviser, the critic, the systematiser of German ideas. He brought to the sciences which he studied a great talent for arrangement, but he was not a creative genius. He gave to human knowledge a didactic form which it had not previously received. It was the order, method, and comparative clearness of his writings that enabled him to reign over the mind of Germany for more than half a century, in a manner which Leibnitz, for want of more system, for want of a vernacular garb for his thoughts, and for want, perhaps, of an equally enlightened tribunal to appeal to, had not attained. It must not be supposed from what has been said that Wolf was a mere echo or copyist of Leibnitz. In many respects he differed from him. Wolf was a kind of eclectic philosopher, and in this he showed wisdom: for unless truth be sought on the principles of eclecticism, an existing age must reject much of the advantage which is to be derived from the history of human thought—its errors and its triumphs. Wolf's eclecticism was not bound by any national or sectarian prejudices. Like Leibnitz himself, he borrowed from the ancients as well as from the moderns, and he did not disdain to cull from the scholastics. We sometimes find him endeavouring to bring Descartes and Leibnitz into union. In his attempts of this sort, he has shown judgment and independence, if he has sometimes failed in the arduous task of reconciling different points of view. It must be admitted that his harmonizing efforts sometimes lead him into heterogeneities and incompatibilities with himself.

Wolf has developed germs of thought, and combined scattered ideas from Leibnitz: he has modified the elements which passed through his hands, and clothed them in a new form. Descartes, Spinoza, and Leibnitz, all more or less employed a method borrowed from mathematics: Wolf aimed at this method in a more rigorous form. In fact, the idea of reducing philosophy to mathematical formulæ was his idol: it was founded on a capital oversight of the essential difference there is between the science of quantity (in which such elements as time, space, and the fixed relations of number are concerned) and the mental and moral phenomena of man in general. Even the example of Spinoza does not seem to have warned our author from this course. The exact sciences are strictly and purely "rational." On the basis of definitions, and with the aid of undisputed axioms, the most extended and intricate combinations become plain by means of a series of transformations in which there is no room for any difference of opinion or point of view. Logic is based on these grounds, and the exact sciences as such are exemplifications of logic. But beyond this domain—in the speculations of psychology and morals—experience has a wide sphere. Their objects are of a mixed and often of an indefinite nature, and they do not admit of being reduced to the well-defined conditions of the mathematical sciences. In order to meet this difficulty, Wolf endeavoured, in various branches of philosophy, to separate the purely rational part from that which belonged to experience, and to reduce all under one system by prolix demonstrations. That he was as successful as he thought himself to be, none of his readers will now admit. The appearance of mathematical accuracy in subjects not mathematical may seem grave and strict, inspiring the expectation of great accuracy; but the appropriateness of the method, beyond the domain of the exact sciences, has now long since been more than questioned. It was, however, popular in Germany under the auspices of Wolf, and it was not till Kant arose that a final blow was given to this dogmatic method of reducing all knowledge. Wolf had at least the merit of putting this method, as an organon of all truth, to the test; and it must be admitted that, however unsatisfactory his procedure for the cure of error may have been, his influence on the entire intellectual development of Germany was very great, and the German language is much indebted to him for being the first to make it the general vehicle of philosophy.

In his *Ratio Prælectionum* our author prefers Aristotle's division of the sciences (theoretic, poietic, and practical) to that of Lord Bacon (history, poetry, philosophy); but proposes his own, which is history, philosophy, and mathematics. Science he defines to be the "process of demonstrating what is asserted." Philosophy he terms the "science of all that is possible and real, and of the why and wherefore of its possibility and existence"—it is the "science of what is and has been, and of that of which a reason can be assigned."* Cause is that which contains within itself a reason for the existence of some other thing. Relatively to the maxim that every thing, change, or circumstance, must have a cause—he states a somewhat qualifying condition, namely, that "what has only a contingent existence must be produced by some efficient cause."† The first thing which ought to occupy the philosopher is *Logic*, or "rational philosophy,"‡ which derives its principles from ontology, or the "science of being *à priori*," and from psychology. Wolf's logic is essentially Aristotelian, though like most of the logicians, he has made his own changes and emenda-

* Logik, *Vorbericht*, § 5. † Ontol. cap. ii.

‡ Vid. *Philosophia Rationalis sive Logica.* 1734.

tions. He treats it in the way of Leibnitz, and defines it the "science which directs the thinking faculties in the search after truth." He divides it into theoretical (with nearly the ordinary heads) and practical logic, which distinguishes and discovers truth, is a basis for criticism, communicates instruction, estimates evidence, and aids in the common duties of life. The criterion of truth in a proposition is that the "predicate may be determined by the notion of the subject." This looks at first like Kant's description of the "analytical proposition," to the exclusion of the "synthetical" one; but on tracing the theory of knowledge as held in the school from Descartes, we see that it was meant to be a general account of propositions. Descartes' criterion of truth was the "distinctness and clearness of the ideas" under which the proposition is couched; and Leibnitz's criterion was the "consistency of the ideas among themselves." The latter view seems hardly to differ from Wolf's, except in words.

Our author's speculations on Truth are much hampered by his artificial method, and by his assumptions respecting the extent to which mathematical principles may be applied in general. His developments are often exceedingly tedious, and his proofs are sometimes not more evident than the proposition to be proved. He has long disquisitions on method, on hypothesis, on the inductions of experience, and the like topics, which are felt by the reader to be not only wearisome but also trite and commonplace. Respecting the connexion of the sciences, we again encounter his rigid abstractions, in his attempts to seek their relations in *à priori* deductions rather than in any more practical and obvious source. In discussing these and other topics, however, he conferred upon his age the benefit of introducing even into popular use a great number of scientific terms which had not previously been generally current.

Many of our readers will be aware* that Leibnitz, while reducing all truth to the two principles of "contradiction," or identity, and of the "sufficient reason" (the former of which included all *à priori* truth, and the latter all other truth), nevertheless maintained that, in the last analysis, even the principle of contradiction might be regarded as falling within the range of the principle of the sufficient reason: for the proposition $(a + b) + (a - b) = 2a$ has a sufficient ground for being believed, from its denial involving a contradiction. Wolf, with less propriety, we think, holds the opposite view, that the foundation of all rational knowledge, even of the principle of the sufficient reason, as well as of every other metaphysical axiom, is the principle

* See our notice of Leibnitz in a former Number.

of contradiction: for, says he, "if a thing had no sufficient reason for its being, something must arise from nothing, which is a contradiction."* To us there appears, here, a confounding of strictly logical with metaphysical truth—the analytical with the synthetical judgment, which were afterwards so well distinguished by Kant.

Our author's division of philosophy is into two general parts—*theoretical*, which relates to knowledge, and *practical*, which relates to action. Under the first head he includes *Ontology*, *Psychology*, *Cosmology*, and *Natural Theology:* under the second, *Ethics*, *Politics*, and the *Law of Nature and Nations*. This classification has to a considerable extent influenced the method of those systems of philosophy which have subsequently appeared among the Germans. We may add, that Wolf tried everywhere to separate the "purely rational" from the "experimental" part of knowledge; but it is generally admitted that he was not very successful in disentangling elements which are so closely blended in all our cognition.

*Ontology*, or *philosophia prima*, relates, after the scholastic fashion, to the doctrine of being, (*Wesenheitslehre.*) And here we find Wolf differing from his great predecessor with respect to the simplicity, composition, and essential nature of substances, a topic abstruse and shadowy enough in the hands of Leibnitz, and not much illuminated by the criticisms of Wolf. Leibnitz said that only simple or uncompounded substances are to be regarded as having *reality* (ὀντως ὀντα): Wolf admitted also compound ones to the honour of this category—and why not? Leibnitz bestowed on each of his monads, or simple unities, a sort of representative force (*Vorstellungskraft*) each being a kind of "mirror of the whole universe:" Wolf denied this,† and rendered the doctrine of the schools less mystical by retaining, as common to all the monads, only the inward efficient energy. Leibnitz's ontology is a pure ideal monism: Wolf's is a dualism of spirit and matter, of simple and compound, of representive or perceptive, and non-representive substances. The universe with him is not a living organization, as Leibnitz maintained, but a mechanism on which force has been impressed. The dualism of Wolf was so far a recession towards the old Cartesianism, and not an onward march from Leibnitz towards the absolute and nihilistic idealism which at last marked the German school in our own times. It is no wonder that Wolf's dualistic ontology is much blamed by some of the later Germans—it was at least two degrees from zero. In regard to the essence or nature of a

* Log. iv. § 5. Metaphysik, iii. § 192. † Ontol. § 64. *Kosmol.* § 598.

thing, we find him mainly agreeing with Leibnitz.* This nature, or essence (*Wesen*) is its intrinsic possibility, the reality being the fulfilment of the possibility.† The essence of the composite is the simple, for the composite can have its cause only in the simple, which renders it possible.‡ We know from reason that there are simple existences, though they never present themselves in experience. Their origin is inexplicable; but they cannot in any natural way perish, nor can they undergo change otherwise than by the alteration of their limits.§

A substance, or a thing subsisting for itself, is that which has the sources of its changes in itself: but a thing which subsists by means of another thing is nothing else than a limitation of the preceding.‖ Now, since the source of changes is called power or force, it is necessary that in everything which subsists for itself there should be a certain power;¶ and changes which take place by means of this power in a thing subsisting for itself are actions of this thing, which have their cause in itself: and through these actions a thing evidences its reality and self-dependence (*Selbständigkeit*), as well as its distinction from other things.** Moreover this power or force must not be confounded with a bare faculty (*Vermögen*), for a faculty marks only the possibility of doing anything: force, on the other hand, is that by which the possibility is translated into reality—that is into a continuous effort, or an action which produces an effect, and which consequently helps a possible into existence (*hilft einem Möglichen ins Daseyn*).†† And since everything which subsists for itself is making a continual effort to change its limits, that is, to alter its condition, and as nothing, and therefore no change can occur without a cause, therefore the preceding change must always contain something out of which the following one has its origin: for in this way only is the course of nature at all conceivable.‡‡

It is evident that closely as Wolf followed the traces of his master in his ontological speculations, he did not hesitate, as he went on, to modify Leibnitz's doctrines according to his own more logical and less imaginative turn of mind. Leibnitz endowed even his lowest order of monads with some kind of perception and appetency. Wolf, as we have seen, was not so indulgent to the plebeian herd as to allow them to be a kind of living "souls" (*âmes*); and by thus distinguishing the higher from the lower class, he evidently receded from the bold advance of his predecessor, who brought them, in some respects, into one

* Monadologie. † Metaphys. cap. ii. § 55. ‡ Ebend. § 76.
§ Ibid. §§ 86, 87, 96, 102, 106—108, 113. ‖ Ibid. § 114.
¶ Ibid. § 115. ** Ibid. § 116. †† §§ 117—120. ‡‡ Ibid. § 128.

category; and Wolf was, so far, apart from the identistical idealism which afterwards reigned in the German school.

*Psychology* (Seelenlehre) is, according to Wolf, either empirical or rational. The former is merely the history of our actual consciousness; the latter is the "science of what is possible (i. e. *à priori*) with respect to minds." The being within us, the soul (*Seele*) is conscious of itself and of other things without itself; and its own existence is immediately certain to every knowing thing as such. The prime activity of the soul is the exercise of the representative faculty (*Vorstellung*), or of the power of forming ideas. From this, as united with consciousness, knowledge arises; and such union of representation with consciousness is called thinking.* The understanding (*Verstand*) is the source of those clear and distinct ideas which are owing to the proper self-activity of the soul. The senses (*Sinne*) and the imagination (*Phantasie*) are the source of direct sensations, and of obscure and confused representations. Reason (*Vernunft*) is nothing more than the faculty of discovering the general connexion of truths by means of conclusions and inferences.† We see, here, that the distinction between understanding and reason, was not first introduced into the German school by Kant, as some have supposed: it is evidently a Leibnitz-Wolfian distinction; but it was not always consistently maintained—not even by Kant himself.

Nothing corporeal can *think*, says Wolf, that is, can by its own activity represent anything to itself with consciousness—nay, it cannot even be passively sensible of anything acting on it, so as to feel the change effected. The power of thought and the faculty of sensation (*Empfindungs-vermögen*) belong exclusively to *soul*, which is placed in entire contrast with body, being incorporeal and simple. All the ideas and sensations which occur to it are only modifications (*Modificationen*) of its own constant and unchanging essence, produced from within or without itself.‡

The souls of men alone, adds our author, are spirits (*Geister*); that is, beings which are simple, having the faculty of representation, endowed with understanding, will, and freedom: consequently they alone are immortal. The souls of animals have, indeed, the faculty of representation, but they have no understanding, freedom, and will: like the simple (uncompounded) points (monads) of inanimate bodies they are not subject to natural decay, but they are not destined to be immortal.§

All the movements of the human soul are dependent on its own peculiar nature, conformably to which it represents to itself

* Metaphys. cap. v. §§ 192, 194.
† Ibid. §§ 194, 277, 282, 284, 368.
‡ Ebend. §§ 222, 738, 742, 784.
§ Ibid. §§ 896, 921, 926.

the objects of the universe; just as all the movements of the body follow from the nature of its own peculiar composition. The fact that these affections of the soul and the body harmonize with each other, is not the consequence of a reciprocal influence between them, nor of the immediate agency of the Deity, nor of occasional causes,* but is the result of a previously-established *harmony* which is not to be ascribed to the mere general principle of the will of God as the Author of nature, but depends on the special principle that every soul always represents to itself the world only according to the constitution of its own organic body, and according to the changes which take place in its organs of sense. Hence those representations and these changes always take place exactly at the same time, without the one being properly caused by the other: we should rather say, that both have their cause in a third thing—namely, the changes which are occurring in the universe itself, which reflect themselves in the body and on the soul.†

Wolf appears to have limited the Leibnitzian doctrine of "pre-established harmony" to the mutual relations of the body and the soul. Leibnitz himself had made it general throughout the universe. The monads had no real agency on each other; none of the objects in the creation exercised any reciprocal influence—not more than two clocks, each independent of the other, one of which is regulated to strike the hour at the moment the other points to it. This harmony is not the result of the immediate and constant agency of the Creator, but of an original arrangement of the parts and forces of the universe, which is a kind of machine set in motion at its creation by the first cause. Of course neither this theory itself, as applied to mind and body, nor any conceivable modification of it, solves any difficulty, such as, for instance, might occur especially to the moralist. It is fair, however, to remark that both Leibnitz and Wolf strenuously maintained the "freedom of the will," and repudiated the form of necessarianism—fatalism—which some have charged upon the doctrine of pre-established harmony,‡ according to which

* The doctrine of "occasional causes" was developed from the Cartesian principles probably by Geulinx of Antwerp, and was fully adopted by Malebranche: it amounted to this—that God is the real agent in all changes; what are usually termed "secondary causes" are only the *occasions* on which he acts.

† Ebend. §§ 765, 979, 368, 786.

‡ This "harmony," unfortunately, seems to have been one source of the *discord* between Wolf and his colleagues in the Senate at Halle, and especially the cause of his incurring the displeasure of the King of Prussia, which led to his banishment, as we have already stated. Euler, in his "Letters to a German Princess," says that when the King was told that Wolf was lecturing to his students on "Pre-established Harmony," he inquired what it meant. Frederic William I. was not possessed of a very subtile understanding, and had no patience for philosophy. One of his courtiers waggishly told him that it was a doctrine according to which they both maintained that the soul acts as it would do if there were no body, the body as if there were no soul. It is not the volition of my soul that moves my arm, or causes the force to act which moves it; both agencies are independent of each other, and both are merged in the original pre-ordained harmony by which they seem, but only seem, to co-operate.

Wolf's *Cosmology* defines the universe to be a series of things finite and changeable, partly contemporaneous, partly successive, and united together in a whole. The rational science, which has for its object the world as a whole, capable of undergoing certain changes, is called by the above names (*Kosmologie—Weltallslehre*).* The changes in the universe are conditioned by the nature of its composition, according to the laws of motion, therefore by its mechanism: hence the universe may be compared to a piece of clockwork or a machine.† In virtue of the general laws of this universe-timepiece, no contingency is imaginable; everything which once has its ground in the series of things which belong to the universe comes necessarily to pass. Still this necessity, says Wolf, is also hypothetical, for the universe might have been different from what it is; various other connexions of things were possible, only they could not have been brought into real existence at the same time with the present arrangements.‡ Wolf, again, does little more than rehearse Leibnitz when he adds, that the constituent parts of the physical world are the bodies (*Körper*) which compose it; the constituent parts of bodies are their simple elements or natural unities (the Leibnitzian monads). The elements or unities have no special magnitudes, and therefore cannot be distinct from each other in quantity or figure, but only by means of powers and qualities; and they really are distinguished from each other, inasmuch as that nowhere in the whole universe one of them is perfectly like another.§

The language of Wolf as to dynamics is a compound of that of Newton with that of Leibnitz. Every body (*Körper*) has a certain amount of innate force (*Trägheits-macht—vis inertiæ*), by which it strives to maintain itself in its place, and to hold its existence and its essence; on the other hand, every body has also a moving power (*Bewegungskraft—vis motrix*), by which it endeavours to change its condition, and to act without itself.‖ The continuity of bodies, and their extension in space, although his Majesty's soldiers were nothing but "*mere machines*, so that if they deserted they could not help it, and were not by any means, therefore, worthy of punishment for it." The King immediately flew into a rage, and ordered Wolf to quit Halle without delay.

* Metaphysik, oder vernünftige Gedanken, u. s. w. cap. 4, § 544.

† Ibid. § 556. ‡ Ibid. §§ 569, 576. § Ibid. §§ 583, 585, 586, 589.

‖ Ibid. § 607. See also Leibnitz's *Monadologie*.

they are composed of simple and inextended elements or unities, arise from this, that their elements are quite distinct from each other, without their unity among themselves being thereby prevented or impaired ; for this unity is founded solely upon the connexion of the condition of each monad with the condition of all the rest.* This Leibnitz-Wolfian doctrine of elements wholly without extension, having no parts and no position in space, but yet constituting the bodies which are extended in space, is a paradox which no ingenuity can solve. Wolf's argument confounds the alleged unity of inextended elements with the property of extension ; it is like saying that though nought or zero is nothing in itself, yet if you heap up an indefinite number of simple noughts you will obtain an arithmetical quantity !

Some of the later Germans complain that Wolf has, in some of these cosmological dogmas, lowered too much Leibnitz's theory of a universe of monadic souls, and almost changed it into a dead mechanism. They allege that he seems chiefly to conceive of the material world as a multiplicity of varying forms, showing themselves in the changes which are evident to our senses, while he almost seems to forget the unity and unchangeableness of the essence of the whole. It is true he makes the unities or monadic elements to have a certain life and activity, but he denied them the perceptive power with which Leibnitz endowed them. He made them, it is alleged, neither material nor spiritual, so that what they are is not plainly evident.† We are not at all disposed further to trouble our readers with these knotty points; we have already dwelt quite long enough on them. We will not enter on the question how far this criticism is just, or whether it is quite consistent with itself. Some of Wolf's cosmological speculations are, no doubt, obscure enough, and there is little here to choose between him and his master. His failure to make intelligible to the speculative intellect of his most acute countrymen who are advocates of idealism what these said "monads" are, speaks for itself. He is complained of for holding the dualism of matter and spirit ; and he is complained of, at the same time and in the same quarters, for making the monads, which are of the essence of either or both, neither one nor the other. The moral of this is the old lesson : it is no wonder that so bright a genius as Leibnitz, and so arrant a plodder as Wolf should equally break down in the attempt to substitute fine-spun theories for real evidence, and to cure man's ignorance by giving loose to the reins of imagination.

* Metaphysik, §§ 604, 605.

† Vid. Rixner's Geschichte, ii. 203, *Anmerk.*—We must here acknowledge the aid we have derived from this author's labours.

With respect to *Natural Theology*, Wolf endeavoured to elaborate the Cartesian arguments from the *à priori* principle of necessary existence, and from the psychological fact of our notion of superior power: but in regard to the principle of the *sufficient reason*, he agreed with Leibnitz, who had maintained that "without this principle we cannot arrive at the proof of the existence of a Deity."* This is basing the grand fundamental truth of religion clearly on the ground of *causation;* and whatever may be said respecting what have been technically named by the later Germans the "ontological," the "cosmological," and the "physico-theological" arguments,† respectively, we are decidedly of opinion that, so far as they have any force, they are all virtually only so many forms of the argument from causation, which is evidently itself founded on the essential constitution of the human mind—an analysis than which nothing can be more ultimate and final. The external universe, says Wolf, as well as our own souls must have a sufficient reason (*zureichenden Grund*) of their existence: now neither of them contains any such reason in itself, for they are not of themselves, and cannot be; therefore the sufficient reason of both must be contained externally to both in a being which requires for its existence no ground or reason beyond its own essence. This being is God.‡

Wolf's further views respecting the nature and attributes of the Divine Being scarcely differ from those of philosophical theologians in general, up to the time when pantheism reduced all theology to an idle dream. God is simple (*einfach*) unchangeable, and unique (*einzig*)—an eternal, infinite, and absolutely self-existent being, the first and the last, before whom nothing was, and after whom nothing can be; a being entirely of and from himself, and independent of all other beings.§ In strong contrast with the dogmas of Spinozism, which so much gave the cue to some forms of the subsequent pantheism of which it was the parent, Wolf held that all that is was created by the Deity, not through mere necessity, but by reason and will. He is the absolute, free, and all-wise Author of all things, and he cannot be the mere "soul of the world" (*Weltseele*—ψυχὴ τοῦ κόσμου), that is to say, he cannot stand in that relation to the universe in which the souls of men and beasts stand to their bodies: for God knows all things immediately, of himself, and not as the soul knows mediately through the body. We must regard him as the purest, the most perfect, the most unlimited spirit, free from all corporeal impediment; a spirit whose understanding is all light and clearness, in which alone all things as to

* Recueil de Diverses Pièces.—*Vid.* our notice of Leibnitz in this Journal.

† *Vid.* Kant's Kritik der r. Vernunft (Rosenkranz), s. 462.

‡ Metaphys. cap. 6, § 945. § Ibid. §§ 947, 948.

their conception and their possibility were always contained, since nothing is possible but in so far as God recognised it from all eternity. From the divine attributes, as those of a being who is not only almighty, but all-wise, all-benevolent, all-perfect, it follows, as Leibnitz maintained in his "Optimism," that among all possible worlds God has created that which is the best.*

Under the head of "Practical Philosophy," we find those subjects treated which are usually comprehended under this name among the Germans: *Ethics, Politics,* and the *Law of Nature and Nations.* Baumgarten, we believe, was the first who added Æsthetics, or the philosophy of the sublime and beautiful, to this division. In his *Ethics,* theoretical and practical, Wolf sets out with the principle that consciousness truly attests man's freedom of choice in all actions of the will. Still he maintains that motives have such a determining power that their effects are inevitable. It is impossible that we should not will what presents itself to us, in our present state of mind, as good, and reject the contrary, as soon as the two objects are clearly apprehended. That which furnishes the motive by which we are determined to volition binds us to action, for without motive we cannot act. Liberty, therefore, is the faculty which man possesses to determine himself according to what appears to him, at the time, the best choice. It is evident that this theory of freedom and volition, as held by Wolf and his predecessor Leibnitz, was similar to that of President Edwards, the great Transatlantic metaphysical divine. It was, in fact, the doctrine of philosophical necessity as much guarded as possible against interference with human responsibility, as we find it expounded in Edwards's great work, "The Freedom of the Will."

Our author, in detailing the moral processes of the mind, remarks that men's sensibilities and ideas are the source of their appetites and their determinations—and this in virtue of the native energy of the soul itself, which has a faculty of desiring as well as of knowing. This faculty of desire, like the knowing faculty, is twofold: inferior and sensuous, which belongs also to the brutes, and the higher or rational form of desire which distinguishes man from the lower creation. The desire (or the aversion) which belongs to sense is an inclination or disinclination of animal nature to a given object, arising out of the idea or feeling of sensuous perfection or imperfection, the former being connected with pleasure and satisfaction, the latter with the absence of pleasure, with dissatisfaction, pain, or with hatred or disgust. On the other hand, rational and intelligent desire or aversion—that which belongs purely to the mind

* Metaphys. §§ 951, 982, 959.

itself—is excited solely by intellectual perfection, fitness, and moral good, or by the want of these, or by their opposites. The actually existing desire or aversion of the individual always necessarily follows the sensuous, or the intelligent and rational ideas which predominate in him: but his freedom of will consists in a man's always determining himself for that which appears to him the best in his existing inclination.*

Wolf's general principle of practical morals is, that every man should, to the utmost of his power, do that which is adapted to render his own condition and that of others as *perfect* as possible. Moral perfection he regards as essentially consisting in the "agreement of the results of a free action with the previous and subsequent conditions of being, according to a law of nature established by the Divine will." Reason is the power by which man takes cognizance of moral rules. Everything which tends to perfect the condition of man as a rational and moral being is *moral good:* whatever makes him more imperfect is *moral evil.* All man's free actions are necessarily either morally good or morally evil, as either conducing or not to the real highest welfare of man (*dem wahren Besten*) present and eternal.† Hence the universal principle of moral action may be reduced to the precept: "As far as lies in thy power, do that which truly makes thee and thy condition, as well as all others and their condition, more perfect, and forbear the contrary." The obligation of this principle, adds our author, lies in the divinity of reason itself, which, according to the will of God who has created us rational beings, we are bound unconditionally to obey.‡ Reason takes cognizance of the conjunctures which arise as consequences of our moral actions; and by the estimate she forms of these consequences she echoes the law of nature on which morality is founded. The beneficial consequences which God has connected with certain free actions constitute *natural reward;* the opposite consequences of other (bad) actions constitute *natural punishment.*§ But as man is a rational being, he is a law to himself, and so far as *reason* prevails, he needs not that rewards and punishments should lie in the perspective. Only let reason be supreme, and he will determine himself to known good and will forbear from known evil. So far as he does this he will be happy; and his happiness consists in a perpetual advance from perfection to perfection.‖

While admitting that all moral good originates ultimately in God, Wolf regards actions as good or bad, intrinsically, and for their own sake, independently of the divine sanction or com-

* Metaphys. §§ 432, 492, 878, 889, 514, 519. † Ibid. §§ 422, 426.

‡ Moral, oder Vernünftige Gedanken von der Menschen Thun und Lassen, §§ 9, 12. § Ibid. §§ 36, 37. ‖ Ibid. §§ 24, 38, 44.

mand. This doctrine of the immutability and necessary fitness of morality is, we think, undeniable; even God himself "does *right*;"* and if we are commanded to obey our parents, the reason is that "it is right."† Our author goes further, and, with many of his successors, argues from the idea of duty, moral fitness, and moral order, that the obligations of morality would still subsist, even apart from the belief in a Deity, that morality is binding, as a law of nature, even on the atheist himself. But as human nature has received from its Author the laws which govern it, he is, in this sense, the prime source of moral law; and he has attached happiness to virtue and misery to vice as a part of the whole economy of causes and effects.

In his discussions on Ethics, Wolf has not only given rules for self-knowledge, but has also, by way of promoting our knowledge of others, introduced some physiognomonical remarks, which were the more ingenious because he anticipated Lavater, who at a later period elaborated the theory of physiognomy, in which, however, he is said to have had less confidence in after-life. Our author's ethical system has been objected to as liable to difficulty on account of a certain vagueness and indetermination attaching to his fundamental idea of "perfection." His system certainly seems to want something more definite in another respect—that which relates to the means of exciting a moral impulse that shall sway the conscience, a faculty which, though so important in the sphere of "practical" philosophy (in the German sense), he has not dwelt on to the extent that might have been desired. His moral philosophy may be said to have a considerable tendency to *endemonism*, the perfection of happiness being apparently made the end of man's existence, rather than that goodness which draws happiness in its train; but Wolf's ethical system would be most unfairly treated if it were confounded with the utilitarianism of later times.

Wolf's *Jurisprudence* naturally partakes of his ethical theories. He considers natural right as resting on the same foundation as moral law, in so far as both tend towards perfection. Every right has its corresponding duty, and right must have duty as its foundation. The aim of all law should be to advance the human species towards perfection, by a constant progression; and if the whole race is thus to go on from perfection to perfection, then the same moral idea must prevail in the commonwealth as in the mind of the private individual; "every one in the community should do that only which the perfection of his own condition and that of others unitedly involves and demands, and should refrain from doing that which would make his own

* Gen. xviii. 25. † Eph. vi. 1.

condition unitedly with that of others more imperfect."* Every man has originally the same rights with every other, in the highest perfection; but in the State every one can only lay claim to a perfection of right conformable to his position, since the very nature of an organic union of individuals implies that, among the several members and their particular callings, the greatest multiplicity and diversity should exist, though without injury to the most harmonious order of the whole. No man, therefore, should either do or forbear doing anything thoughtlessly, or without an express purpose; as every rational purpose will subserve the last and highest end of social life, the perpetually growing perfection of the human race.†

Wolf has been blamed for having comprehended under jurisprudence certain rules which belong only to morals, and with having too often subjected the principles of natural right to the maxims of the Roman law. We are not disposed to detain our readers with a detail on this point; but to whatever extent our author may here have erred from love of theory, there is no doubt that he was one of those writers who gave a new impulse to the study of jurisprudence in Germany by the elevated aim which he assigned to it, the place he demands for it among the moral and political sciences, and the general interest which he contrived to throw around it.

Our voluminous and indefatigable author also treats copiously of *Political Philosophy*, under which he discusses the domestic, social and governmental relations, the rights of the sovereign, the executive authority, and international law, including the rules of war. He lays the foundation of political science, as applied to civil communities in the maxim: "Do whatever the common safety and the common good require." That is the best government which most efficiently tends to this issue. He considers the monarchical form as most calculated to fulfil the above axiom, though he admits that it has certain drawbacks. His notions on government are not always quite consistent with themselves—certainly not always such as to suit our English ideas: and this we need not regret; for he denies to subjects, as such, the right of examining what the general interest demands, and reserves this right to the sovereign. He does, however, limit by the laws the right of the sovereign to do what he deems the public weal to require. Political economy is another topic under this head, and here Wolf handles various points relating to the well-being of society in general—such as the wealth and power of nations, and the means of their advancement. But many of his views on these subjects show that, in his day, political

* Politik, oder Vernünftige Gedanken vom gesellschaftlichen Leben der Menschen, im gemeinen Wesen, § 1.

economy was quite in its infancy, and that his ideas respecting it were chiefly influenced by what then existed, and not so much by a wide survey of the future destinies of society. Still, Wolf did good service by including among subjects which called for philosophical treatment, matters which up to that time had been considered as limited to the cabinets of princes, and hardly open to public discussion. He, at all events, secured a place for the political sciences which should open the way to their impartial and thorough investigation.*

Those among the Germans who have taken the least favourable view of Wolf's labours have represented his philosophy as consisting merely of such fragments from the system of Leibnitz as were most capable of a popular exposition, the more strictly speculative parts being less brought into notice; so that the more recondite points have been too much ignored. The modern idealists regard Wolfianism in the light of a popular eclectic dogmatism, couched under an artificial imitation of the mathematical method, though the author himself sometimes admits that mathematics and philosophy are very different sciences; since mathematics can demonstrate the general conception of the pure form out of every intuition of the single object;† while philosophy, on the other hand, can render the sensuous appearance an object of intelligence only out of the pure conceptions which belong to the supersensual being—soul or mind. The ideal school regards Wolfianism as by no means satisfying the demands of science, and as having obtained the popularity which it acquired in all the seats of learning in Germany solely from its meeting one want, at least, the want of some regular system: it was therefore accepted in the absence of a system of a higher order, and it produced no inconsiderable revolution in the republic of letters. Wolf may be said to have spent his life

* M. Degérando, an elaborate historian of philosophical systems, praises Wolf's "practical" philosophy, for "the vast extent of its plan, its general harmony, its noble tendency, its abundant promises: its point of departure is human freedom, perfection is its aim, nature is its type, disinterestedness its condition, the connexion of rights and duties its result." But Degérando thinks that in the execution, Wolf often disappoints the hopes he had raised in the minds of his readers. He is also one of those who view Wolf's and Leibnitz's doctrine of volition as tending to an objectionable necessity. "Elle (La Philosophie Pratique de Wolff) trompe trop souvent dans l'exécution les espérances qu'elle avait fait naître. Ainsi la liberté s'évanouit sous l'efficacité des motifs déterminants, dont les effets, aux yeux de Wolff, sont inévitables. Aussi a-t-il partagé les reproches dirigés contre Leibnitz, et a-t-il été accusé, comme celui-ci, d'introduire une sorte de nécessité dans l'empire de la volonté humaine."—*Histoire Comparée*, tom. viii. p. 31.

† That is, in German phrase, the properties belong to the general conception of the pure form (*schema*), which can never be reduced to intuition (*anschauung*), the properties, for instance, of the plane *triangle:* but any particular empirical intuition, any plane triangle, of any form or size, will suffice as the means of the general demonstration.

in explaining and defending the principles of his school; for he was ever ready to enter the polemical arena against its adversaries. The rigid order and the close concatenation of his method was an imposing feature of his writings, and from him may be traced the practice of treating every branch of knowledge in mathematical forms which became current throughout Germany. Croon, Kelsh, Stellwaag, Wasser, Feyerlin, Hagen, and others, all wrote in favour of this method. It was applied to theology, to the instruction of youth, and to the teaching of Hebrew: in short, it became general. Poppe, Hismen, and Basedow, at length, put a check to it by their remonstrances.

The difficulty which we have had to encounter in the attempt we have made to give some account of Wolf's views, may be seen from the following passage of Brucker:

"Wolf possessed a clear and methodical understanding, which by long exercise in mathematical investigations, was peculiarly fitted for the employment of digesting the several branches of knowledge into regular systems. The lucid order which appears in all his writings generally enables his reader to follow his conceptions with ease and certainty through the longest trains of reasoning. But the close connexion of the several parts of his works, together with the vast variety and extent of the subjects, renders it impracticable to give a summary of his doctrines."*

We will add a brief quotation from Tennemann, the well-known historian of philosophy, who (after speaking with approval of Wolf's definite method, order, precise distinctions, and improved terminology,) remarks:

"The errors of his philosophy consist in his taking mere thinking as his point of departure; overlooking the difference between the formal and the material conditions of thought; his regarding philosophy as the science of the possible so far as it is possible; making the principle of contradiction the ultimate principle of all human knowledge; in his placing mere ideas and verbal distinctions at the commencement of every inquiry; his drawing no limit between rational and experimental knowledge; his confining the activity of the mind to the phenomena of perception; and in his neglecting to distinguish the peculiarities which separate mathematics and philosophy both in their form and matter."†

The above criticism is for the most part correct: we may state, however, that it is taken from the Kantian point of view, which was destined soon to become almost the exclusive one in Germany. The Wolfian philosophy reached its culminating point while its author was Professor at Marburg, from 1723 to 1740. A reaction had shown itself before his death, in 1754.

* Historia Critica Philosophiæ: vid. *Wolfius.*

† Grundriss, s. 425.

It had, indeed, previously begun to wane, from various causes—the overwrought formalism of its demonstrations, its dogmatic attempts to answer all objections, the pedantry of many of its advocates who tried to reduce the simplest truths to logical demonstration, the introduction of Locke's writings into Germany, the eclectic spirit which ensued; but the rise of Kant, the greatest of the German metaphysicians, and the rapid progress of his philosophy, finally overthrew it.

Wolf was much addicted to the exact sciences; but he made no discovery. His chief merit, here, was as a teacher. His course of mathematics long remained the most complete ever published in Germany; but in this science, the most concise of all by its very nature, he did not fail to manifest his tendency to prolix diffusion. Into philosophy he introduced many new terms of technical meaning from the Greek language, which have been retained by his successors. His German works are usually considered as the best-written; and the language is indebted to him for the facility he has shown it to be capable of, in expressing by German compounds the Latin terms and phrases which had before been employed. This coinage of new words, however, renders a vocabulary sometimes desirable to the uninitiated reader; and this aid has been rendered by the author, at the end of such of his works as seemed to require it. His Latin works are not much to be praised, either for his choice of words, for his adherence to the classic sense, or for general excellence of style.

We close by stating that we are not aware of any edition of Wolf's whole works having been published since his death. The edition of 1739 came out, in octavo, at Breslau. Ludovici wrote a digest of his philosophy, and Hartmann published an introduction, but we have not met with either of these works. Meissner composed a philosophical lexicon, for the explanation of Wolf's system from his German writings. Biographies of him were written by Gottshied and Büsching.

